# The Characteristics of PCDD/F and PCB Occurrence and the Effect of Age in Matched Tissues of Cattle and Sheep from Southern Italy

**DOI:** 10.3390/toxics14040348

**Published:** 2026-04-21

**Authors:** Roberta Ceci, Gianfranco Diletti, Giampiero Scortichini, Ettore Franco, Angelo Pellegrino, Iain R. Lake, Alwyn R. Fernandes

**Affiliations:** 1 Istituto Zooprofilattico Sperimentale dell’Abruzzo e del Molise G. Caporale, via Campo Boario, 64100 Teramo, Italy; r.ceci@izs.it (R.C.); g.scortichini@izs.it (G.S.); 2 Dipartimento di Prevenzione-Azienda Sanitaria Locale di Taranto, Contrada Rondinella, 74123 Taranto, Italy; 3 School of Environmental Sciences, University of East Anglia, Norwich NR4 7TJ, UK

**Keywords:** paired liver and muscle, dioxins, food contamination, aryl hydrocarbon receptor (AhR), CYP enzymes

## Abstract

Toxic environmental contaminants, such as polychlorinated dibenzo-*p*-dioxins and furans (PCDD/Fs), and polychlorinated biphenyls (PCBs) occur differentially in animal tissues. This study examined paired liver and muscle tissues from the same animals, reducing the uncertainty inherent in other studies that source tissues from different animals. Investigations were carried out on cattle and sheep from two separate herds in Southern Italy. As all animals experienced the same environmental impacts, husbandry, and feed regimes, contaminant distribution between tissues would result from physiological considerations, which would also allow for better examination of the effects of age. In both investigations, PCDD/F and PCB concentrations were significantly higher (*p* < 0.01) in the liver relative to muscle. A characteristic occurrence pattern showed PCBs dominating the combined toxic equivalence (TEQ) by >95% in cattle tissues and 78% and 67% in sheep muscle and liver, respectively. A majority of liver samples exceeded regulated maximum limits, and the herds were excluded from the food supply. Subsequent regional monitoring showed regulatory compliance of cattle/sheep meat and liver, but prominence of PCB-TEQ persisted. Concentrations of both contaminants declined strongly in the tissues of both species with increasing age of juveniles but stabilized in older animals (>one year in sheep; 2/3 years in cattle). Although weight gain might partly account for this pattern, the initial decline may also relate to inadequate levels of CYP enzymes in the youngest juveniles, but this would need to be confirmed in both species by targeted toxicokinetic studies during this perinatal period. The expression of these detoxifying enzymes is reported to rise rapidly with increasing postnatal age in many animal species, including sheep.

## 1. Introduction

Some animal tissues are known to retain relatively high levels of polychlorinated dibenzo-*p*-dioxins and furans (PCDD/Fs) and polychlorinated biphenyls (PCBs), which are toxicologically and chemically related classes of food and environmental contaminants [1,2]. The laterally substituted congeners of both these contaminant classes are able to assume planar configurations which are chemically and biologically stable [3,4]. The characterization of the risk to health from exposure to these PCDD/F and PCB congeners is based on multiple effects, ranging from pre-carcinogenesis, tumor progression, immunosuppression, reproductive toxicity, endocrine disruption, etc. At extreme levels of human exposure, mortality through acute hepatocellular degradation and cirrhosis has been documented [5,6]. This extreme effect is related to the historical use and manufacture of PCBs or accidental discharges of PCDD/Fs. Given the current state of awareness of the risk, the phasing out of PCB production, and the regulatory control on inadvertent emissions of both PCDD/Fs and PCBs, these levels of exposure are now unlikely. However, in the absence of occupational or accidental exposure, dietary intake is the predominant exposure pathway in both humans and animals. Following ingestion, PCDD/Fs and PCBs are partitioned from the digestive system and distributed via the blood and lymphatic systems to various receptor organs, including the liver and adipose tissue [7]. In common with other halogenated aromatics, PCDD/Fs and PCBs are lipophilic and are thus strongly retained in mammalian lipid tissues, variously as subcutaneous fat, visceral fat, and intramuscular fat, or in lipid-rich organs.

Despite several years of environmental and food regulation (PCDD/Fs and PCBs were regulated in food and animal feed within the EU since 2002 through a system of maximum occurrence limits [8] and regular monitoring of food concentrations), there is very little literature relating to PCDD/F and PCB occurrences in corresponding tissues from the same animals [9,10,11]. Most monitoring studies in support of these regulations investigate representative samples of food and animal feed that originate from different sources of supply. Thus, as a relevant example, beef obtained from a retail outlet and categorized as carcase meat may be sourced from a location with a distinctive contamination profile, while beef liver (sold in the same outlet or location), which is categorized as offal, may be sourced from a different location with a different contamination profile. Other differences in relative tissue contamination levels can arise if the samples are collected at different periods in the growth cycles of the animals, from locations with different levels of contamination, different feed intakes, and also from the sex of the animal (in older females, contaminant levels can vary after birthing and lactation). Consequently, studies that report on the occurrences of these contaminants in meat and liver that are sampled independently fulfil a general surveillance requirement relating to food safety but may not accurately reflect the relative distribution in tissues from the same animal.

In much the same way as noted in other modern industrialized countries, animal products, including meat, liver, milk, butter, eggs, etc., from Italy have been reported to be contaminated by PCDD/Fs and PCBs [1,12]. As a result of increasing awareness, incidents, such as the Belgian contamination incident, and also as a result of the National residue control monitoring programs in Italy, a number of instances of elevated PCDD/F and PCB levels in foods of animal origin were uncovered [13]. The sources of contamination and the occurrence levels in animal products have varied, depending on the location in which the animals were raised. Products such as cow’s milk from Alpine pastures in the north of Italy showed expectedly lower levels of PCBs (sum of 13 congeners), ranging from 4.6 to 7.4 ng/g lipid [14]. Milk from Lombardy and Emilia-Romagna showed mean PCDD/F and dioxin-like PCB (DL-PCB) toxic equivalent (TEQ) concentrations of 1.26 pg TEQ/g fat (mean value of 0.34 pg PCDD/F TEQ/g) and 9.30 ng/g fat for NDL-PCBs (non-dioxin-like PCBs) [15], comparing well with the alpine data. Some well-publicized instances of contaminated foods were the result of localized environmental contamination. For example, although dioxin contamination in the Campania Region in Southern Italy was noted in 2001 and resulted from the non-compliance of two ewe milk samples, the more publicized incident of buffalo milk contamination in the Campania Region in Southern Italy [13,16] was uncovered in 2008 as part of a wider monitoring plan involving the screening of a number of environmental pollutants. In the most contaminated animals, World Health Organization—TEQ (WHO-TEQ_2005_) levels as high as 87 pg PCDD/F TEQ/g lipid for buffalo milk (with a corresponding concentration of 15.9 pg DL-PCB TEQ/g lipid) from Caserta province, Campania, in the south of the country were recorded [17]. In Northern Italy, bovine fat and liver taken from 28 contaminated animals from farms proximate to a historical PCB manufacturing site near Brescia in Lombardy showed elevated levels (PCDD/F plus PCB TEQ ranged from 30 to 81 pg/g lipid in fat and from 107 to 138 pg/g fat in the liver). PCBs contributed 90% and 81% of the sum TEQ in fat and liver, respectively, and the authors attributed the contamination to a commercial PCB mixture [18].

Similarly, a median sheep liver TEQ (PCDD/F + PCB) of 9.9 pg/g lipid vs. 2.21 pg/g lipid for cows liver was reported for samples collected from the Piedmont region of Northwestern Italy [1] compared to a median TEQ value of 63.8 pg/g fat in ovine/caprine livers from farms in the Taranto region in 2008/2009 [19]. More recent reports based on national sampling showed mean PCDD/F and PCB levels of 0.82 pg TEQ/g lipid in beef [20] and 0.88 pg TEQ/g whole (wet) weight (ww) (11.7 pg TEQ/g lipid) in sheep liver [12].

This report is based on two investigations carried out on cattle and sheep that were raised in the Apulia region in Southern Italy in two proximate provinces, Taranto and Lecce. The aim of the study was to examine PCDD/F and PCB tissue distributions in liver and muscle without the uncertainty of differentially sourced tissues, and secondly, to investigate the effects of age on these distributions using animals from the same herds. Uniquely, the muscle tissue and liver of the animals were taken from the same animals. The combination of the two sampling characteristics, i.e., using animals from the same herds and using matched tissues from the same animal, provides greater reliability to the objectives of this report. Using animals from the same herds minimizes variations from environmental impacts, differences in husbandry, veterinary inputs, and diet that arise when animals are sourced from different locations. The paper also supplements the data from these two investigations with the results of more recent surveillance of PCDD/Fs and PCBs in these products, particularly those produced in the South of Italy.

## 2. Materials and Methods

### 2.1. Description of the Study Area and Sampling of Tissues

In both investigations, paired muscle meat and liver tissues were sampled from the same animals, 16 cattle and 28 sheep. The cattle were raised on a farm near Taranto, a coastal city in Apulia, Southern Italy. Taranto has relatively high levels of industrialization, including the largest steel plant in Italy, an oil refinery, a cement plant, and thermoelectric plants. The sheep were raised at a farm in the proximate province of Lecce, where the main industries are wine and olive oil processing and the manufacture of pottery and glass. The sheep pasture was close to an industrial area and the local MSW incinerator. Both sampling areas were known to have at least historical contamination—a local municipal solid waste (MSW) incinerator in close proximity to where the sheep were raised in the Lecce province and the known generally higher contamination levels in the Taranto region—as a likely result of industrial activity that has been reported in other studies [17,21].

Sixteen cattle, eleven female and five males, were selected, with ages ranging from nine to 176 months (just under 15 years), with a median age of 36 months. Similarly, 28 sheep, mostly female, were selected with approximately equal numbers of animals from five age groups—lambs at less than three months, lambs from three to six months, and ewes after their first, second, and third lambing (up to 48 months). Sampling was conducted between 2009 and 2016. Muscle meat and liver were collected from each animal by the official veterinarian at the point of slaughter. Samples were stored in polyethylene containers, immediately refrigerated for transport to the laboratory, and subsequently frozen at −18 °C until analyzed.

As a temporal comparator to the above two investigations, samples of cattle and sheep liver and muscle tissue that were collected as part of national food surveillance over the last 7–8 years were also analyzed by the laboratory. These samples (cattle: 72 of liver, 287 of muscle; sheep: 19 of liver, 87 of muscle) represented locations in the South of Italy, but unlike the two investigations above, the liver and muscle were not matched, and samples were collected from diverse locations.

### 2.2. Analysis, Data Processing, and Data Quality

The analytical methodology used for the determination of PCDD/Fs and PCBs has been reported in detail [19,20,22] and has been used in other studies [12,21]. In brief, each tissue sample was individually homogenized, allowing a representative aliquot (equivalent to 3 g of fat for muscle, approximately 5 g for liver) to be taken for analysis. Analytical standards, both native and ^13^Carbon (^13^C)-labeled surrogates of all regulated [23] congeners (seventeen PCDD/Fs, eight dioxin-like PCBs, and six NDL-PCBs), were obtained either from Cambridge Isotope Laboratories, Tewksbury, MA, USA, or from Wellington Laboratories Inc., Guelph, ON, Canada. The samples were internally standardized with ^13^C-labeled surrogates of the analytes, mixed with sodium sulfate, and extracted using accelerated solvent extraction [22]. The extracts were purified using an automated, modular Fluid Management System (FMS, Billerica, MA, USA), yielding separate eluates for PCBs and PCDD/Fs, which were concentrated and sensitivity standardized with ^13^C-labeled standards. These extracts were analyzed by gas chromatography coupled to high-resolution sector field mass spectrometry (HRGC-HRMS, using a DFS instrument supplied by Thermo-Fischer Scientific, Waltham, MA, USA).

The concentration data obtained from the above process were analyzed statistically to provide conventional summary parameters (minimum, maximum, mean, median, and standard deviation and error of mean where appropriate) for individual tissues within each study. Prior to this statistical treatment, concentrations of PCDD/F and dioxin-like PCB (DL-PCB) congeners were converted to toxic equivalents (TEQs) using TEF_2005_ (toxic equivalent factors) values as specified in regulations [23]. Similarly, as per regulatory requirements, the six non-dioxin-like PCB (NDL-PCB) congeners were summed to provide an indication of the cumulative PCB concentration. The measured tissue concentrations of PCDD/F and PCB TEQ are expressed as pg/g fat; the corresponding summed NDL-PCBs are expressed in ng/g fat. Both liver and muscle tissue data sets from the two investigations were examined separately for outlying values using the Grubbs test. The distribution of the concentration data within each set was also examined using Mann–Whitney U tests in order to establish the significance of any difference between the paired tissue concentrations. Relationships between animal age and tissue concentrations were studied using Pearson correlation.

All analyses were carried out by the Italian National Reference Laboratory for halogenated Persistent Organic Pollutants (POPs) following the regulated analytical guidelines for determining PCDD/Fs and PCBs in food [23], which specify quality parameters, such as LOQ and analyte recovery (between 60 and 120%). As part of the associated quality control measures, the analysis of the study samples included regular procedural blanks and reference materials (BCR-607, and also, performance test (PT) materials, which have been assigned consensus values), which were assessed to be satisfactory and within the required limits. Procedural blanks were used to establish the LOQs (0.01 pg/g fat for PCDD/F TEQ as well as for PCB TEQ and 0.01 ng/g fat for NDL-PCBs), according to EU guidance [24]. Most of the analyzed congeners were detected during analysis, apart from 2,3,7,8-TCDF, 1,2,3,7,8,9-H_6_CDF, and 1,2,3,4,7,8,9-H_7_CDF, which were frequently below the LOQ. In addition to using validated and accredited (to ISO17025 standards [25]) methodology, the laboratory is also obliged to participate twice yearly in performance testing on PCDD/Fs and PCBs to confirm the reliability of analytical data that it provides on food and animal feed. The results from this external assessment (conducted by the European Union Reference Laboratory for POPs), carried out during the period of these investigations and since, demonstrate the reliability of data, achieving Z-scores between ±2.

## 3. Results and Discussion

A revised set of toxic equivalency factors (TEFs) for summarizing PCDD/F and DL-PCB concentrations as TEQs has recently been proposed [26], but it has not yet been officially endorsed by any regulatory body, so TEQ_2005_ values have been reported. The main effect of this revision would be to reduce the TEQ values for both PCDD/Fs and, particularly, PCBs. While the choice of TEF values used would have an impact on food safety, the main topics of this paper are relative tissue distributions and the effect of animal age on contaminant uptake, both of which are comparative evaluations that are unlikely to be affected by the choice of TEF value.

### 3.1. Cattle Study

The tissue concentration data is summarized in Table 1. PCDD/F TEQ concentrations showed a relatively narrow range for both liver (1.04–4.72 pg/g fat) and muscle (0.15–0.89 pg/g fat) in comparison to PCBs, which showed >8 fold difference in occurrence for both TEQs (14.8–131 pg/g liver and 6.5–54 pg/g muscle) and for NDL-PCBs (71–596 ng/g and 30–283 ng/g in liver and muscle, respectively). PCBs clearly made a dominant (>95%) contribution to the TEQ in both sets of tissues, as seen for the mean and median values. Although the range of PCB concentrations was relatively large, data were, in general, evenly distributed around the mean (skewness of 1.07).

The main characteristic of the data set was clearly the difference between paired liver and muscle tissue concentrations from the same animals—liver concentrations were consistently and significantly (*p* < 0.01; Mann–Whitney U tests) higher. Additionally, the data were also characterized by the absolute concentrations of PCB TEQ (and by association, PCDD/F plus PCB TEQ) and NDL-PCBs. PCDD/F TEQ concentrations were unremarkable, not only within the current data set, i.e., the contribution of PCDD/Fs to the sum TEQ was on average 1.8% for muscle and 5.3% for liver, but also in comparison to other similar literature data [10,27,28]. As a result of the expected contamination, the entire herd of cattle was excluded from the food chain, but an evaluation of the concentrations against the relevant EU regulations [8] showed that none of the PCDD/F TEQ values breached the maximum level (ML) threshold (0.3 pg/g wet weight [ww] for liver and 2.5 pg/g fat for muscle tissue compared to the highest liver concentration of 0.19 pg/g ww and highest muscle concentration of 0.89 pg/g fat measured in this study). However, the majority of samples (all except two liver samples) exceeded the MLs for combined PCDD/F and PCB TEQ and for NDL-PCBs [8].

### 3.2. Sheep Study

In contrast to the results of the cattle study, PCDD/F TEQ in both sheep liver and muscle tissue (Table 1) ranged over an order of magnitude (0.42–102 pg/g fat in liver and 0.18–1.9 pg/g fat in muscle tissue). The PCB TEQ range was not dissimilar (0.94–117 pg/g fat) in liver but was considerably wider in muscle tissue (0.15–10.6 pg/g fat). The NDL-PCB concentrations ranged over almost two orders of magnitude for liver and an order of magnitude for muscle tissue (2.75–219 ng/g fat in liver and 4.53 and 45.9 ng/g fat in muscle). The contribution from PCBs to the sum-TEQ was approximately two-thirds in the liver but more than three-quarters in the muscle tissue, as seen by the mean and median values. The values of these parameters also suggest that the data was relatively skewed towards higher concentrations (skewness = 2.05). PCB concentrations (TEQ and NDL-PCBs) in two of the 28 animals could be considered as outliers by the classical definition (>mean + 2SD) and also as determined by the Grubb test.

As expected, liver concentrations of PCDD/Fs and PCBs were considerably and significantly (*p* < 0.05) higher (>25 fold) in the liver than in the corresponding muscle tissue but surprisingly, despite the proximity of the waste incinerator, which can generally be considered as a source of PCDD/Fs [29,30], PCBs were still the dominant contributor to the sum TEQ in most animals (mean ratio of PCB TEQ to PCDD/F TEQ was 2.5 in the liver and 4.7 in the muscle tissue). Prior to the study, some non-compliances relative to the EU maximum limits in food had been found in the milk and tissues of some sheep from the farm, so at the time of the study, the animals were removed from the food chain, as they were considered unsafe for human consumption. In the case of the cattle, none of the muscle tissue of the animals in the study exceeded the PCDD/F ML of 2.5 pg TEQ/g fat, but about 20% of the study animals would have exceeded the combined ML (4.0 pg TEQ/g fat) for PCDD/Fs plus PCBs, and the majority of the liver samples would have exceeded the corresponding ML values for liver [8].

### 3.3. Recent Routine Surveillance Data

In order to provide context to the contamination levels seen in the above investigations, the concentrations were compared against contemporary data on sheep and cattle tissues sourced from the regional food supply. These more recent concentration data not only included locations in the provinces of Lecce and Taranto but also other cities in Southern Italy. An important distinction to be made with these recent data is that, unlike the two investigations described above, the concentration data were not derived from matched tissues from the same animals but rather from samples taken during routine food surveillance. Nonetheless, these data, representing more than 460 liver and muscle samples collected in the last seven years to 2023, provide a more current indication of PCDD/F and PCB concentrations, as well as the relative distribution of PCDD/Fs and PCBs in cattle and sheep tissues taken from the south of Italy.

The data from these routine surveillance studies is summarized in Table 2. In order to allow easier comparability, the data are presented on a fat weight basis for both tissues (liver data, which are now required to be reported on a ww basis [shown in grey font in the Table] for regulatory purposes, were recalculated to fat weight, as described in the table footnote).

The summarized data in Table 2 provide reassurance that contemporary food supplies of liver and muscle from cattle and sheep, obtained as part of national surveillance sampling plans, from across the south of Italy are considerably less contaminated than those seen in the animals from the two investigations described above. In fact, none of the reported concentration levels exceeded the maximum limits (see footnote in Table 2) specified in the amended Commission Regulation of 2023 [8]. The ratio of contaminant concentrations in liver relative to muscle tissues in these contemporary samples remains similar to those seen in the two investigations, although, as described earlier, the comparison is less robust due to the different provision and sources of the sample tissues. However, in common with the two investigations on cattle and sheep, the relative contributions of PCDD/F and PCB to the summed TEQ continue to show the dominance of PCBs in the muscle tissues of both species, i.e., the ratios are approximately 3:1 for cattle and 1.4:1 for sheep in favor of PCB TEQ. For the liver of both species, the ratio lies between 0.8 and 0.9, which is higher than those reported in other studies [10,27,28,31,32]. Thus, the relative contribution of PCBs to the TEQ seen in the two investigations on cattle and sheep from contaminated farms is also reflected in the general supply of cattle and liver products across the south of Italy. This dominance of PCBs over the TEQ in cattle and liver tissues is also seen more widely across Italy, as reported earlier [12] from the Italian national monitoring studies (2013–2016) for regulated food, in general.

### 3.4. Discussion

**Figure 1:**
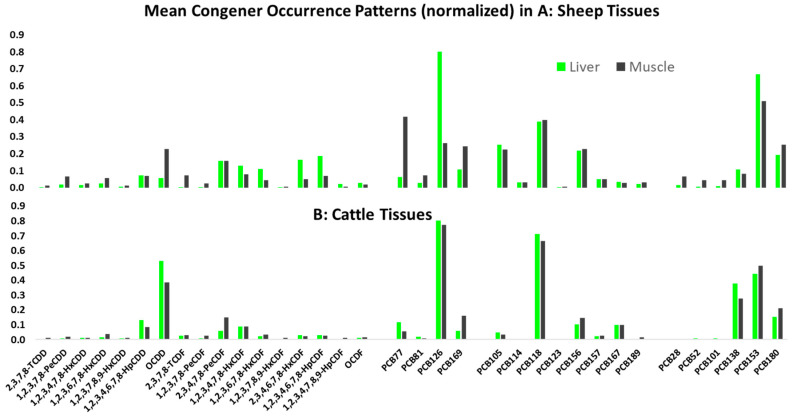
Mean congener occurrence patterns in sheep and cattle liver and muscle tissue. Occurrence within groups (PCDD/Fs, DL-PCBs separately as non-ortho-substituted, mono-ortho-substituted, and NDL-PCBs) is normalized because of different concentration ranges.

The main observation on the congener patterns in cattle and sheep tissues is the difference between the two species as seen in Figure 1 (in order to aid visualization, the occurrences are normalized within each contaminant group—PCDD/F congeners are normalized to the sum of PCDD/F congeners; Dl-PCB congeners are normalized in two groups, i.e., to the sum of non-ortho-substituted DL-PCBs and to the sum of mono-ortho-substituted DL-PCBs; NDL-PCB congeners are normalized to the sum of NDL-PCBs). For both PCDD/Fs and PCBs, cattle tissues show the dominance of a smaller number of congeners, and in general, for the PCBs, liver and muscle show similar patterns of occurrence. Sheep tissues, on the other hand, show a much fuller distribution of congener occurrence with different patterns for muscle and liver. For example, PCDDs are relatively dominant in muscle but not in the liver. These patterns suggest different rates of congener uptake and metabolism between these two species of ruminants, as noted in other studies [33]. The patterns may also be influenced by the amount of soil ingested during feeding, as both cattle and sheep consume significant amounts of soil during grazing [34].

The fundamental observation from the concentration data presented (Table 1 and Table 2) from the two investigations on matched tissues and from the later surveillance samples is the elevated levels of PCDD/Fs and PCBs in liver relative to muscle tissue. This is evident in all three data sets and has also been reported in the literature for a number of animal species, including sheep, cattle, pigs, and humans [1,10,27,28,35,36,37]. The occurrence of PCDD/Fs and PCBs in both tissues is influenced by their lipophilicity, which promotes association with tissue fat. In the liver, in addition to this association, the occurrence of PCDD/Fs, PCBs, and other similar lipophilic contaminants is also augmented through the physiological function of this organ in synthesizing and directing the metabolism of the various types of fats (triglycerides, phospholipids, fatty acids, cholesterols, etc.) that are required for energy or are redirected to storage in fat depots [38]. However, association with fat on its own does not explain the steep differential in muscle and liver concentrations seen, for example, in sheep tissues. The de-toxification function of the liver, in which hepatic enzymes bind to these contaminant ligands, which are thus sequestered [33,39], also contributes to the higher liver burden. This has been reported for different species, including rodents, which showed elevated liver concentrations relative to adipose tissue following exposure to increasing doses of 2,3,7,8-TCDD [39], but also in pigs and sheep [33]. Therefore, in practice, many farmed species raised for food production, such as cattle, sheep, pigs, and dairy cows, have been reported to show this characteristic [1,27,28,35,36] under normal husbandry conditions and background levels of contamination. However, this may not apply to all farmed species. Chickens, for example, showed similar concentrations of combined PCDD/F and PCB TEQ at ages of market readiness in liver and muscle tissues when reared under uncontaminated conditions [10,40]. Even in more contaminated environments, such as E-waste dismantling sites, the reported median concentration of PCDD/F + DL-PCB TEQs of chicken meat and chicken liver were 4.92 and 1.66 pg TEQ/g ww, respectively [41]. Similarly, in a study on laying chickens fed on contaminated feed, TEQ levels were higher in the fat and eggs of the animals compared to the liver [9]. However, at very high levels of intake, such as those seen during the Belgian dioxin contamination incident, prolonged exposure (12 weeks) could result in relatively higher levels in chicken liver [42].

Another defining characteristic of these tissue data sets on cattle and sheep is the relatively high contribution of DL-PCBs to the summed TEQ. The contribution was approximately 98% and 95% on average for cattle muscle and liver, respectively; the corresponding values for sheep were 78% and 67%, respectively. The literature reports on sheep and cattle liver from different parts of Europe, such as Germany, Poland, the UK, and Greece [10,27,28,32], suggest that the contribution from DL-PCB TEQ is either smaller or comparable to PCDD/F TEQ when samples were taken from uncontaminated sources. Although a few other countries have reported DL-PCB contributions that exceed those of PCDD/Fs, the marked dominance of PCB TEQ seen in these samples is uncommon.

While it is clear that the samples from the cattle and sheep investigations were taken from farms that were known to have suffered contamination, the sources of the contamination at both farms remain unclear. In the case of the cattle, the contamination profile is heavily biased towards PCBs, with unremarkable levels of PCDD/Fs (the highest PCDD/F TEQ values were 0.89 pg/g fat in muscle and 4.72 pg/g fat [0.11 pg/g ww] in liver). For the sheep samples, however, the profiles were expected to be dominated by PCDD/Fs because of the proximity of the farm to an MSW Incinerator, a likely PCDD/F source. The maximum PCDD/F TEQ values were 1.9 pg/g fat in the muscle tissue samples and 102 pg/g fat in the liver samples (equivalent to 2.12 pg/g ww which exceeds the regulated maximum limit) but curiously, despite the obvious influence of the MSWI source, PCB TEQ still dominated, contributing 67% in the case of liver and 78% for muscle to the summed TEQ (Table 1). DL- and NDL-PCB congener profiles in the sheep liver and muscle samples were similar to those in other studies, which looked at sheep tissues from uncontaminated backgrounds [38], which suggests that while the MSWI may have been a probable source [43] of the elevated PCDD/Fs, it was unlikely to have contributed to the high PCB concentrations.

There have been reports of elevated PCB concentrations in animal products in Southern Italy, reflecting the profiles in the sheep tissues reported here, but it is unclear what the source of the PCB contamination might be. However, based on available knowledge of potential sources or agricultural practices in the region, some reasonable hypotheses could be proposed. One possibility is the historical production and use of technical PCB mixtures, but production sites were only known in Northern Italy. Recently, high PCB levels in goat’s milk were reported [44], likely resulting from the earlier, widespread practice of illegal dumping and disposal by open burning of toxic urban and industrial waste produced all around Italy, in the Campania region of Southern Italy, over a number of years. The pattern of elevated PCB TEQ in the cattle study also resembles that reported in the literature for meat from young animals (veal) on a farm in Switzerland [45], with an average PCB contribution of >95% to the TEQ (comparing well to the mean 95% contribution in the present cattle study). Further investigations to elucidate the possible source of the contamination indicated that PCB-containing paint on the walls of the cattle housing had degraded over time and contaminated the straw that was used as feed [45]. In the cattle study here, the water supply, pasture grass, and conventional commercial feed that were provided to the animals were investigated for PCDD/F and PCB content but contained low contaminant levels. The cattle housing material was unfortunately not investigated at the time, but as the buildings dated back to the period after the First World War, it is possible that some materials in the structure could have contained PCBs, as has been reported by other studies investigating contamination in farm animals [45]. It is worth noting, however, in addition to the illegal disposal of urban and industrial wastes [44] and resulting high levels (mean—170 ng/g NDL-PCBs) in goat’s milk, relatively high levels of PCBs have also been reported for milk and dairy products from the same region [11]. These foods, sampled over six years (1005 milk and 70 dairy products from 2013 to 2018) from Taranto, showed a similar dominance of PCB TEQ over PCDD/F TEQ, with mean values for milk of 0.28 pg/g fat for PCDD/F and 1.1 pg/g of combined PCDD/F and PCB TEQ. Corresponding values in the milk products were 0.21 pg/g fat and 0.75 pg/g fat, respectively, representing 3–4-fold higher concentrations of PCBs. Spatial analysis concluded that higher levels of PCBs were associated with areas closest to industry [11,46]. Two other recent studies on the PCDD/F and PCB contents of Italian animal produce, based on national sampling, also showed relatively higher PCB TEQ in beef/veal (0.59 pg/g PCB TEQ vs. 0.2 pg/g PCDD/F TEQ) and in sheep muscle tissue (0.52 pg/g fat PCB TEQ vs. 0.22 pg/g fat PCDD/F TEQ) [12]. Later, Ceci et al., 2022 [20], reported 0.67 pg/g fat and 0.15 pg/g fat for PCB and PCDD/F TEQ, respectively, in beef and 1.51 pg/g fat and 0.29 pg/g fat for PCB and PCDD/F TEQ, respectively, in sheep milk.

The comparative evaluation of the contamination patterns in these two investigations against those seen in contemporary samples (Table 2) provides a wider spatial insight, as these samples represent locations in the south of Italy. These recent samples show compliance with the regulatory requirements and hence a greater measure of safety of these products, but characteristically, the muscle tissues continue to show dominance of PCB contamination over PCDD/Fs in this region, which as mentioned, is distinctive in comparison to other countries in Western Europe, including Greece, Germany, UK, Ireland, the Netherlands, Poland etc. While the reasons for this pattern are not entirely clear (historically, Italy was a significant producer and user of technical PCB mixtures—e.g., Fenclor, Pyralene, and Apirolio series, but so were the UK, Germany, Poland, etc.), it appears to be distinctive in terrestrial animal products in some countries, including those in immediate proximity to Italy, e.g., Switzerland, Austria, France [47,48], and of course, in Southern Italy. A single recent study on meats from the south of Italy [49] did show the pattern observed in other European countries, but the samples were taken from supermarkets, and because of national (or even international) distribution and the pooling of samples, the samples may not be representative of the local produce.

### 3.5. Effects of Age

**Figure 2:**
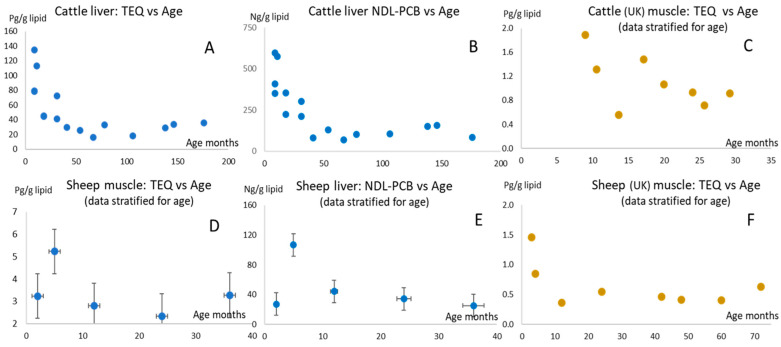
The effects of animal age on tissue concentrations (PCDD/F + PCB TEQ and NDL-PCBs) in cattle (upper charts) and sheep (lower charts). The youngest study cattle were 9 months; the youngest sheep were markedly juvenile at ~2 months. (**A**,**B**) show trends in contaminant concentrations in cattle liver with age. (**D**,**E**) show trends in contaminant concentrations in sheep tissues with age. (**C**,**F**) show trends in TEQ concentrations in cattle and sheep muscle from a UK study [50] with age.

In cattle, the distribution of contaminants between organs, and in particular, liver and fatty tissues, may also be influenced by the age of the animals. In the two sheep and cattle studies, the concentrations of PCDD/F and DL-PCB TEQ and NDL-PCBs in both sets of tissues appear to decline with age in both cattle and sheep (Figure 2—upper plots relate to cattle, lower plots to sheep). For NDL-PCBs, the highest concentrations were seen in the youngest animals but declined sharply by more than two-thirds between the second and third year (12 to 36 months) of age (Figure 2B). Following this decline, concentrations did not appear to vary greatly (up to 14.5 years or 174 months). A similar, although smaller decline (a little more than half), between 12 and 36 months was seen for TEQ (Figure 2A). Similar data from studies in the UK on cattle and sheep raised conventionally until market readiness [50,51] is plotted for comparison. The cattle in this UK study (Figure 2C) were monitored over a shorter period and were sourced from different locations along the course of a river but showed a similar decline in concentrations over 10 to 30 months.

The study on sheep covered ages from approximately two to forty months (3.5 years). The data for individual animals was stratified to provide mean concentrations for discrete age groups. The youngest animals showed relatively lower concentrations at two months, but at three–four months, a sharp increase was followed by a similar decline with age (Figure 2D,E), as observed for the cattle. Comparison to the data from a UK study [50] on sheep reveals a similar decline with increasing age, although the youngest animals monitored in the study were a little older at three months of age (Figure 2F).

The initial analysis of the data [50] that examined the relationship between age and contaminant concentrations in sheep reported no evidence of a trend as the animals aged. The rapid but varying body weight gain in individual animals may introduce differences in contaminant tissue burdens through dilution. Indeed, both TEQ and NDL-PCB concentrations appear to vary with increasing age when data for individual animals were plotted. However, in the comparative plot of the same data shown in Figure 2F, the variations arising from different sampling locations (variations from environmental impacts and different husbandry methods from farms across the UK), as well as natural biological variation between individual animals, were reduced by stratifying the contaminant concentrations for discrete age periods.

Fetal body burdens supplemented by postnatal lactational transfer are the most likely reason for the relatively high contaminant concentrations seen in both muscle and liver tissues in the juvenile animals. Following the onset of weaning, contaminant intake fluxes are likely to change as the diet is more varied. However, these relatively elevated levels may also arise because of the lower capacity in juveniles for metabolism of contaminants, as described earlier [52]. This effect was observed in juvenile cattle during a transgenerational tissue distribution study on PCDD/Fs and PCBs [53]. Earlier, the effect was also variously noted in studies on contaminant burdens in polar bears and marine mammals [54,55], and for pesticide burdens in humans [56]. In a similar way, the capacity of the youngest calves and lambs in this study to detoxify contaminants is likely to be considerably lower than older animals because the basic physiological and metabolic processes in very young animals would tend to focus on growth, increase in body weight, development of organs, etc., rather than on the development of hepatic enzymes that would allow binding and detoxification of contaminants. It is unclear at what age this capacity overcomes the threshold that allows adequate levels of enzyme development that would enable effective detoxification. The sharp decline seen in sheep contaminant concentrations (both TEQ and NDL-PCBs—see Figure 2D–F) may coincide with the developmental phase of these enzymes, as well as with the onset of weaning (which would gradually remove the contribution from maternal transfer) and rapid increases in bodyweight, which would dilute tissue concentrations. Notwithstanding the exact age at which this occurs, it is clear that the youngest animals are exposed to higher levels and effects of PCDD/Fs and PCBs. A very similar situation is seen when TEQ and NDL-PCB concentrations in liver and muscle from the cattle study are plotted against age (Figure 2A–C). Here, the youngest age at which samples were taken was nine months (which again, may coincide with enzyme development and also with the natural weaning age of cattle), so the sharp decline in concentrations resembles that observed for the sheep tissues.

It has been reported in a study on sheep that some hepatic CYP proteins, such as CYP2A and CYP2C, were not present in the fetus or in newborn animals [57], who are thus unable to metabolize the substrates, but this ability increases with advancing postnatal age [58]. The absence of these enzymes correlates with the reports of underdevelopment of CYP enzymes in human neonates (specifically, human CYP1A2 is expressed increasingly after infancy and abundantly after the first year) and in other species [55,59,60,61] and indicates that very young lambs have a reduced ability to metabolize CYP ligands, such as PCDD/Fs and DL-PCBs. It is not unreasonable, given the very similar observations for cattle (Figure 2), to hypothesize a similar condition for very young calves. However, this hypothesis would need to be supported by toxicokinetic studies specifically targeting the perinatal period in bovines, and also, for confirmation, in sheep and other species. Thus, the patterns in juvenile tissue burdens with increasing age probably arise from a combination of the effects of weight gain, weaning, and the development of CYP enzymes, but the relative contributions of these factors are not known. Future toxicokinetic study designs could include this investigation.

## 4. Conclusions

The use of paired tissues has conclusively shown that the concentrations of PCDD/Fs and PCBs (DL-PCBs and NDL-PCBs) were significantly (*p* < 0.01) higher in the liver of sheep and cattle relative to the muscle tissue. This observation concurs with other reports on higher contaminant loading in the liver of some species and was also seen during more recent food surveillance reported in this study. As expected, contamination in the animal tissues from the sheep and cattle study was relatively high, with a number of samples exceeding regulated limits, but food surveillance of similar tissues over the last seven years, sourced from Southern Italy, showed regulatory compliance.

DL-PCBs dominated the TEQ profile in the paired samples from both investigations, with low PCDD/F contribution. This dominance was lower but persisted in sheep tissues despite the proximity of the animals to an MSWI and was also seen in the muscle tissues of later food surveillance samples. It is, therefore, likely to be a characteristic of the PCDD/F and PCB contaminant profile in cattle and sheep from the region. Future research that targets sampling and detection of potential pollution sources in the region would be helpful in reducing similar contamination.

PCDD/F and PCB concentrations showed an initial strong decline in both liver and muscle tissue with increasing age of the juveniles, but they stabilized over time in older animals. This trend may in part be due to the increasing gain in weight of the animals and the onset of weaning, but the initial decline may also relate to the increasing development and expression of CYP enzymes in the youngest animals. In many animal species, including humans, the expression of these enzymes, which facilitate the metabolism of ligands, such as PCDD/Fs and DL-PCBs, is reported to increase rapidly with increasing postnatal age. Future toxicokinetic studies would be required in order to elucidate the relative contributions of weight gain, weaning, and the development of CYP enzymes to the observed tissue patterns.

## Figures and Tables

**Table 1:** Summary of PCDD/F TEQ, PCB TEQ, and NDL-PCB concentrations in muscle and liver tissues in Italian cattle and sheep.

**Cattle Study**	Liver	Muscle	**Liver**	**Muscle**	**Liver**	**Muscle**	Liver	Muscle	Liver	Muscle
*n* = 16 animals	^1^ Concentrations: TEQ in pg/g fat, NDL-PCBs in ng/g fat									
	Minimum		**Median**		**Mean**		Maximum		Skewness	
PCDD/F TEQ	1.04	0.15	**2.09**	**0.29**	**2.38**	**0.38**	4.72	0.89	0.9	1.1
PCB TEQ	14.8	6.49	**36.5**	**17.8**	**49.5**	**24.0**	131	54	1.3	0.9
ΣPCDD/F + PCB TEQ	15.8	6.64	**38.6**	**18.2**	**51.9**	**24.3**	135	55	1.3	0.9
SUM NDL-PCB	71	30.4	**184**	**58**	**244**	**108**	596	283	1.0	1.1
**Sheep Study**										
*n* = 28 animals	Minimum		**Median**		**Mean**		Maximum		Skewness	
PCDD/F TEQ	0.42	0.18	**13.6**	**0.51**	**19.8**	**0.70**	102	1.9	2.6	0.9
PCB TEQ	0.94	0.15	**25.6**	**2.28**	**37.5**	**2.68**	117	10.6	1.4	2.6
ΣPCDD/F + PCB TEQ	1.4	0.79	**39.2**	**3.00**	**57.3**	**3.36**	219	12.5	1.8	2.7
SUM NDL-PCB	2.75	4.53	**29.1**	**10.9**	**47.6**	**13.5**	219	45.9	2.2	2.2

^1^ Concentrations–upper bound, fat weight basis. EU max limits [8] for muscle in pg/g fat are 2.5 (PCDD/Fs), 4.0 (PCDD/Fs + PCBs) & 40 ng/g fat (NDL-PCBs); for cattle liver in pg/g ww, are 0.3 (PCDD/Fs), 0.5 (PCDD/Fs + PCBs) & 3.0 ng/g ww (NDL-PCBs); for sheep liver in pg/g ww, are 1.25 (PCDD/Fs), 2.0 (PCDD/Fs + PCBs) & 3.0 ng/g ww (NDL-PCBs).

**Table 2:** PCDD/F and PCB concentrations in sheep and cattle tissues sampled recently as part of routine food surveillance (liver and muscle tissue are not paired).

**Cattle** **(*n* = 72 liver & 287 muscle)**	TEQ ^1^ Concentrations in pg/g fat; NDL-PCB concentrations in ng/g fat													
	Minimum			**Median**			**Mean**			Maximum			Std. deviation & (Mean error)	
	Liver ww	Liver	Muscle	Liver ww	Liver	Muscle	Liver ww	Liver	Muscle	Liver ww	Liver	Muscle	Liver	Muscle
PCDD/F TEQ	0.02	0.55	0.01	0.04	1.04	0.09	0.05	1.31	0.12	0.18	4.68	0.64	0.03 (0.004)	0.09 (0.006)
PCB TEQ	0.00	0.04	0.01	0.04	0.91	0.29	0.05	1.20	0.36	0.19	4.94	2.22	0.04 (0.004)	0.28 (0.02)
ΣPCDD/F + PCB TEQ	0.02	0.58	0.02	0.08	1.95	0.38	0.10	2.52	0.48	0.37	9.61	2.86		
SUM NDL-PCB	0.09	2.39	0.13	0.36	9.22	1.46	0.48	12.41	1.77	1.33	34.55	9.22	0.33 (0.04)	1.28 (0.08)
**Sheep** **(*n* = 19 liver & 81 muscle)**														
PCDD/F TEQ	0.03	0.54	0.03	0.10	1.82	0.14	0.14	2.50	0.20	0.43	7.81	1.46	0.094 (0.02)	0.21 (0.02)
PCB TEQ	0.00	0.03	0.002	0.09	1.63	0.19	0.11	2.04	0.29	0.35	6.36	2.00	0.09 (0.02)	0.31 (0.03)
ΣPCDD/F + PCB TEQ	0.03	0.58	0.03	0.19	3.45	0.33	0.25	4.55	0.49	0.78	14.17	3.46		
SUM NDL-PCB	0.09	1.63	0.12	0.44	7.99	1.23	0.53	9.61	2.05	1.23	22.34	11.68	0.35 (0.08)	2.2 (0.24)

^1^ Liver concentrations on a fat weight basis were calculated from ww data using the median liver fat contents of 3.85% for cattle and 5.51% for sheep. EU max limits [8] for muscle in pg/g fat are 2.5 (PCDD/Fs), 4.0 (PCDD/Fs + PCBs) & 40 ng/g fat (NDL-PCBs); for cattle liver in pg/g ww, are 0.3 (PCDD/Fs), 0.5 (PCDD/Fs + PCBs) & 3.0 ng/g ww (NDL-PCBs); for sheep liver in pg/g ww, are 1.25 (PCDD/Fs), 2.0 (PCDD/Fs + PCBs) & 3.0 ng/g ww (NDL-PCBs).

## Data Availability

The original contributions presented in this study are included in the article. Further inquiries can be directed to the corresponding author.
